# Perceptions and practices of rehabilitation specialist nurses in fall management: a qualitative study

**DOI:** 10.3389/fpubh.2026.1749446

**Published:** 2026-01-29

**Authors:** Heli Zhang, Jianfen Luo, Xiaotian Zhang, Yuting Jiang, Xiaoyu Sun, Qi Tang, Xin Wang, Baohua Li

**Affiliations:** 1 Department of Rehabilitation Medicine, Peking University Third Hospital, Beijing, China; 2 Department of Nursing, Peking University Third Hospital, Beijing, China; 3 School of Nursing, Peking University, Beijing, China; 4 Institute of Sports Medicine, Peking University Third Hospital, Beijing, China

**Keywords:** fall management, patient safety, qualitative research, rehabilitation nursing, risk assessment

## Abstract

**Introduction:**

Falls are a critical challenge in rehabilitation nursing, often leading to severe injury and prolonged recovery. Rehabilitation specialist nurses play an essential role in fall prevention; however, they encounter difficulties in accurately identifying risks, personalizing interventions, and collaborating effectively within interdisciplinary teams. This study aimed to explore the perceptions and practices of rehabilitation specialist nurses in fall management and to identify their strategies, challenges, and recommendations for optimizing fall prevention and intervention in rehabilitation settings.

**Methods:**

An exploratory qualitative study was conducted. Semi-structured, face-to-face interviews (30–60 min) were conducted with 20 rehabilitation specialist nurses from tertiary hospitals, each with at least 2 years of fall management experience. A piloted interview guide with four open-ended questions focusing on risk perception, prevention strategies, challenges, and improvement suggestions was used. Data were analyzed via content analysis using NVivo 14 until code saturation was achieved.

**Results:**

Content analysis identified four main themes: (1) specialized nurses' sensitivity to falls; (2) comprehensive fall risk assessment; (3) system-wide participation in fall prevention; and (4) timely evaluation of fall management effectiveness.

**Discussion:**

Rehabilitation specialist nurses were found to contribute significantly to fall prevention through multidimensional assessment and personalized interventions. Integrating rehabilitation specialist nurses' expertise in dynamic functional assessment into standard fall prevention protocols is crucial for improving patient safety. Healthcare institutions should leverage their expertise to establish structured prevention protocols, incorporate real-time monitoring, and promote interprofessional cooperation, thereby enhancing the effectiveness of fall management in rehabilitation settings.

## 1 Introduction

Fall management is a crucial component of rehabilitation nursing practice, particularly for older patients, stroke survivors, individuals with spinal cord injuries, and those with other chronic conditions. Falls can cause severe physical injuries and negatively impact psychological wellbeing and social participation (1, 2). Rehabilitation nurses play a important role in fall prevention and management, as their expertise directly influence patient recovery and quality of life (3, 4). However, existing research indicates that rehabilitation nurses still face numerous challenges, including insufficient identification of risk factors, lack of personalized intervention, and limited interdisciplinary collaboration (2, 3).

In recent years, with the continuous advancement of rehabilitation medicine, fall management strategies have gradually shifted from single physical interventions to multimodal approaches, incorporating behavioral adjustments, environmental modifications, patient education, and technological aids (5, 6). Nevertheless, the effectiveness of these strategies is often influenced by nurses' expertise, patient compliance, and healthcare resource allocation (7, 8). Additionally, significant variations exist across different healthcare settings (e.g., acute care, long-term care, and community rehabilitation), highlighting the importance of standardized training and evidence-based practice (9, 10). With their specialized background, Rehabilitation nurses demonstrate greater expertise and sensitivity in fall management, making them key voices in practice.

The core roles of rehabilitation specialty nurses in fall management include fall risk assessment and screening, as well as the design and implementation of multimodal intervention strategies. Risk assessment tools such as the Morse Fall Scale and Berg Balance Scale are widely applied, but their effectiveness relies on nurses' professional judgment and clinical experience (3, 11). For example, stroke patients require careful evaluation of balance dysfunction and visuospatial neglect (2, 12). However, the limitations of risk assessment cannot be overlooked. Some nurses may over-rely on tool scores while neglecting individual patient differences (e.g., cognitive status or behavioral habits) (7, 13). For instance, while amputees face prosthetic and gait challenges not addressed by generic assessment tools (5, 7). Therefore, a comprehensive evaluation must integrate medical history, functional status, and environmental context to ensure accuracy.

Rehabilitation specialist nurses need to integrate multiple strategies in fall interventions, including physical training, behavior modification, and environmental adjustments. Physical training (e.g., balance exercises and strength training) is the cornerstone of fall prevention, but its effectiveness depends on the intensity and personalization of the training (6, 12). For example, virtual reality technology (e.g., Xbox Kinect) has been used to improve gait and balance in Parkinson's patients, with its interactivity and engagement significantly enhancing patient participation (6). Behavioral interventions focus on changing patients' risk behaviors and improving self-efficacy. Studies show that patients' insufficient awareness of fall risks or overconfidence (e.g., the mindset of “I won't fall”) are major contributors to falls (4, 5). Nurses can enhance risk awareness through educational programs (e.g., the “Safe Recovery” initiative) and encourage preventive measures (e.g., using assistive devices or avoiding high-risk activities) (10). Additionally, environmental modifications (e.g., reducing home hazards) are particularly important in community rehabilitation but are often overlooked (1, 3).

Nurses in fall management face more challenges and obstacles. Firstly, the limitations in nurses' professional knowledge pose a significant challenge. Although the importance of fall management is widely recognized, some rehabilitation nurses lack in-depth understanding of fall risk factors for specific populations (e.g., spinal cord injury or cognitive impairment patients) (14, 15). For example, patients with cognitive communication disorders (CCD) may struggle to accurately describe their balance issues due to communication difficulties, leading nurses to underestimate their fall risk (15). Additionally, nurses' insufficient proficiency in applying emerging technologies (e.g., telerehabilitation or wearable devices) may limit the innovation of interventions (8, 16). To overcome the limitations in nurses' professional knowledge, it is crucial to clarify the knowledge they need to master and their awareness of their own limitations.

Secondly, the psychological state and behavioral patterns of patients are crucial to the success of fall interventions. Some patients reduce their activity due to fear of falling (“post-fall anger” or “passive acceptance”), which exacerbates functional decline (5, 17). Additionally, cultural background and level of social support can influence patients' acceptance of interventions. For example, Malaysian stroke survivors tend to rely more on family caregivers while neglecting professional rehabilitation advice (1). This places higher demands on nurses, who must use motivational interviewing and personalized goal-setting to help patients overcome psychological barriers and establish positive behavioral patterns (17, 18). Furthermore, insufficient interdisciplinary collaboration may lead to fragmented interventions. Nurses need to work closely with physical therapists, occupational therapists, and physicians, but unclear role boundaries or poor communication can weaken team effectiveness (9, 19). As a highly skilled and specialized group, rehabilitation nurses must explore and implement effective management strategies for key aspects and critical areas of fall prevention.

## 2 Methods

### 2.1 Study design

This study employed a qualitative research design using semi-structured interviews to explore the perceptions and practices of rehabilitation specialist nurses in fall management. This study adopts an interpretivism, emphasizing the exploration of social phenomena through understanding participants' subjective experiences and meaning-making processes. Researchers prioritize the meanings participants assign to their actions and contexts, striving to interpret these meanings through interactions and interviews to gain deeper insights into fall management practices. Researchers are able to reflect on how their clinical backgrounds and prior assumptions about fall prevention influence data collection and interpretation. To ensure a standard of reporting qualitative research findings, we followed the consolidated criteria for reporting qualitative studies (COREQ) guidelines.

### 2.2 Setting and participants

Participants were recruited using purposive sampling. The research invitation was posted in the rehabilitation specialist nurse WeChat group. Inclusion criteria: Holding certification as a rehabilitation specialist nurse from the Chinese Nursing Association; a minimum of 2 years of clinical experience; at least 2 years of direct involvement in fall management. Exclusion criteria: Nurses not currently on duty; nursing interns. Interviews were conducted during participants' non-working hours, either face-to-face in a quiet room or through video conferencing. No monetary incentives were provided to participants. Code saturation was achieved after 20 interviews. No new codes or subcategories emerged beyond the first 18 interviews, and two additional interviews confirmed this. The study has been approved by the Medical Research Ethics Committee of Peking University Third Hospital (ethics approval code: IRN00006761-M2023787). All participants provided informed consent.

### 2.3 Data collection

Face-to-face semi-structured interviews were implemented by two trained researchers. Interview guide was developed by rehabilitation professionals with qualitative experience and was piloted with two participants (Table 1). It included six open-ended questions related to perceptions prevent and manage of fall risks for specialist nurses. Researchers arranged convenient places for the participants to conduct interviews. Interviews were recorded by audio recorder. Field notes were also taken to capture non-verbal cues and contextual details. After the interviews, researchers wrote reflective journals to track how their personal characteristics affected data collection and analysis. Transcripts were returned to participants for correction. Each interview lasted approximately 30–60 min and was audio-recorded with the participants' consent.

**Table 1:** Advanced rehabilitation nurse interview guide.

**Question number**	**Question Content**
Question 1	How do you define a patient's “fall risk”? Among rehabilitation patients, which factors make you particularly vigilant about potential falls?
Question 2	Do you believe a single fall incident could alter a patient's rehabilitation goals or treatment plan? If so, please describe specific scenarios.
Question 3	In your daily practice, what are the most frequently implemented fall prevention measures? Based on your experience, which improvement strategies do you think could significantly reduce fall risk?
Question 4	If you were to design a fall management protocol, what innovative elements would you prioritize?
Question 5	Do you have any additional critical insights or recommendations regarding fall management to supplement the above?

### 2.4 Data analysis

Data were collected and analyzed concurrently. After no additional issues were identified, the codebook began to stabilize, which was recognized as code saturation, and stopped data collected (20). Following verbatim transcription, the transcripts were analyzed using content analysis and managed by the software of Nvivo (21). The first author started by reading transcripts several times, then broke them down into smaller and more manageable meaningful units, followed by coding, creating categories, and grouping them under higher-order themes (22). The corresponding author provided support and refined the themes. Other researchers provided some suggestions for data analysis. All researchers discussed problems encountered during data analysis weekly to reduce bias. All interviews were conducted, transcribed, and analyzed in Chinese, with codes, categories, and themes developed in the original language. Key findings and quotations were translated into English by a bilingual researcher and verified against the transcripts by another, with discrepancies resolved through discussion and minimal adjustments made to ensure clarity while preserving original meanings.

### 2.5 Trustworthiness

To enhance the trustworthiness of the findings, the study adhered to the criteria of credibility, transferability, dependability, and confirmability. Credibility was ensured through prolonged engagement with the data and member checking. Transferability was supported by providing detailed descriptions of the context and participants. Dependability was achieved by maintaining an audit trail of the research process. Confirmability was ensured by reflexivity, with the researchers documenting their assumptions and biases throughout the study.

## 3 Findings

### 3.1 Demographic information

A total of 20 advanced rehabilitation nurses participated in the study, including 1 men and 19 women. Ages ranged from 25 to 44 years, with a mean of 33.85 years. The majority of interviewees held bachelor's degrees (95%). Most nurses were from rehabilitation, orthopedics, and neurology departments, with further demographic details presented in Table 2.

**Table 2:** The characteristics of participants.

**Number**	**Age**	**Gender**	**Education**	**Professional title**	**Department**
N1	36	Female	B	Nurse-in-charge	Department of Neurology
N2	30	Female	B	Nurse-in-charge	Operating Room
N3	42	Female	B	Nurse-in-charge	Department of Rehabilitation Medicine
N4	42	Female	B	Nurse-in-charge	Department of Rehabilitation Medicine
N5	30	Male	B	Nurse practitioner	Department of Orthopedics
N6	25	Female	B	Nurse practitioner	Department of Neurology
N7	27	Female	B	Nurse practitioner	Department of General Practice
N8	27	Female	B	Nurse practitioner	Department of General Practice
N9	37	Female	B	Nurse-in-charge	Department of Rehabilitation Medicine
N10	44	Female	B	Nurse-in-charge	Department of Rheumatology and Immunology
N11	31	Female	B	Nurse practitioner	Department of Orthopedics
N12	43	Female	B	Nurse-in-charge	Department of Orthopedics
N13	42	Female	B	Nurse-in-charge	Department of Neurology
N14	34	Female	B	Nurse practitioner	Department of Rehabilitation Medicine
N15	35	Female	B	Nurse practitioner	Department of Neurology
N16	33	Female	B	Nurse-in-charge	Pediatric Intensive Care Unit
N17	30	Female	A	Nurse practitioner	Department of Orthopedics
N18	29	Female	B	Nurse-in-charge	Department of Orthopedics
N19	30	Female	B	Nurse practitioner	Department of Orthopedics
N20	30	Female	B	Nurse-in-charge	Department of Rehabilitation Medicine

Education, bachelor's degree; A, associate degree.

Qualitative analysis revealed four major themes that were consolidated as follows from the nurse's viewpoint: (1) specialized nurses' sensitivity to falls; (2) comprehensive fall risk assessment; (3) system-wide participation in fall prevention; and (4) timely evaluation of fall management effectiveness.

### 3.2 Theme 1: specialized nurses' sensitivity to falls

#### 3.2.1 Knowledge—familiarity with fall risk factors

Most specialized nurses reported a thorough understanding of fall risk factors, including specific medications, lack of adaptive environments, absence of caregivers, and characteristics of high-risk populations.

“One type of pain patch can cause orthostatic hypotension in patients, so we pay special attention to medications that may increase fall risks.” (N10)

“After being admitted to the ward, patients are often in an unfamiliar environment, which significantly increases their risk of falling.” (N4)

“Patients with prolonged illnesses often experience caregiver fatigue, and their families may not take fall prevention seriously.” (N19)

“We focus on high-risk populations and strengthen management during night shifts and shift handovers when staffing is limited.” (N5)

#### 3.2.2 Belief—the significance of fall management

Specialized nurses emphasized that falls can cause irreversible and severe harm to patients, underscoring the critical importance of fall management.

“For patients who have undergone hip or knee replacement surgery, a fall could lead to readmission, prolonged hospitalization, compromised recovery, and increased medical costs.” (N18)

“Older patients are particularly vulnerable; a fall could result in fractures, exacerbating their existing conditions.” (N11)

#### 3.2.3 Belief—falls are preventable but not eliminable

Specialized nurses believe that while falls can be prevented, it is impossible to completely eliminate them.

“Our fall management is not always comprehensive, and falls can occur across all age groups. While they can be prevented and mitigated, they cannot be entirely eradicated.” (N17)

“Nurses should develop foresight—identifying high-risk factors or behaviors, such as patients attempting to get out of bed independently, and proactively addressing them.” (N10)

#### 3.2.4 Practice -prioritizing high-risk fall factors

Specialized nurses focus on high-risk factors in postoperative orthopedic patients to prevent falls, such as assisting patients during their first attempt to get out of bed, ensuring proper use of assistive devices, providing personalized education, and managing critical time periods.

“We always support patients during their first attempt to get out of bed post-surgery, and proper use of assistive devices is essential.” (N5)

“We use specialized fall risk assessments and patient communication to evaluate cognitive function, awareness, and personality, determining their ability to cooperate in fall prevention.” (N3)

“Patients may overestimate their recovery and attempt activities beyond their current capabilities, significantly increasing their fall risk.” (N4)

“Patients with relatively good self-care abilities may overestimate their mobility, performing actions unsuitable for their condition, which also increases fall risk. Additionally, our educational efforts may sometimes fail to fully resonate with patients.” (N2)

### 3.3 Theme 2: comprehensive fall risk assessment

#### 3.3.1 Holistic fall risk evaluation

Specialized nurses believe that, in addition to standardized assessments, fall risk evaluation should incorporate patients' awareness of falls and specialized assessments, such as muscle strength, joint mobility, and nutritional status.

“Assessment tools can accurately determine the level of fall risk a patient faces.” (N6)

“I feel that the assessment tools are incomplete—they are too rigid and may not capture all the factors contributing to a patient's fall risk.” (N2)

“Beyond fall risk assessment tools, we also evaluate patients' balance and mobility” (N11).

“Specialized nurses can also assess patients' nutritional status as part of fall risk evaluation.” (N20)

“Through patient interviews, we conduct a comprehensive assessment of fall risk, including age and history of previous falls.” (N2)

#### 3.3.2 Frequent and dynamic fall risk assessment

Fall risk assessments need to be dynamic, with frequency adjusted based on risk levels and changes in the patient's condition.

“Assessments are conducted regularly and dynamically. If a patient's condition changes or they are prescribed new medications, their fall risk score and level are updated.” (N13)

“We increase the frequency of assessments for high-risk patients.” (N14)

“As long as a fall risk exists, assessments are conducted during every shift.” (N13)

### 3.4 Theme 3: system-wide participation in fall prevention

#### 3.4.1 Individual level—strengthening patient fall prevention

At the individual level, personalized fall prevention education is essential, and active participation of patients and their families in fall risk management is required.

“After assessment, specialized nurses identify specific fall risk factors and tailor their educational efforts accordingly.” (N13)

“We encourage family members to participate, providing supervision and guidance.” (N12)

“Patient involvement is crucial for effective fall prevention.” (N4)

#### 3.4.2 Hospital level—normalizing fall prevention management

At the hospital level, it is crucial to strengthen fall prevention training for nurses and raise fall prevention awareness among all healthcare staff, while also creating a safe ward environment and encouraging multidisciplinary participation in fall prevention.

“We collaborate with cleaning staff to ensure floors are not overly wet and that warning signs are placed after cleaning.” (N17)

“We mark the rooms of high-risk patients with visible signs, so all staff are aware and can provide additional assistance and frequent checks.” (N5)

#### 3.4.3 Societal level—reducing the burden of an aging population

At the societal level, falls represent a significant public health concern, and effective fall prevention can reduce disability, mortality, and economic losses, thereby making fall prevention a public health priority.

“Falls can lead to decreased self-care ability, prolonged bed rest, and complications, increasing the financial and caregiving burden on families.” (N8)

“In an aging society, falls pose a fatal risk to older patients, so fall prevention should be a collective effort across the entire hospital.” (N4)

“Falls should be a societal focus, as they are a major cause of disability, loss of independence, and death among the older.” (N6)

### 3.5 Theme 4: timely evaluation of fall management effectiveness

Timely evaluation of fall management effectiveness helps determine the success of prevention strategies and identify areas for improvement.

#### 3.5.1 Fall prevention as a quality control priority

“Our nursing department places a strong emphasis on fall prevention. Each month, we conduct safety incident discussions across all departments to analyze fall cases and implement improvements.” (N16)

#### 3.5.2 Assessing patient awareness of falls

“After a period of intervention, we reassess patients to determine if their awareness and behavior regarding fall prevention have improved.” (N6)

#### 3.5.3 Implementing corrective measures after falls

“When a fall occurs, we analyze the root cause and implement corrective measures to prevent recurrence.”(N17)

#### 3.5.4 Evaluating nurses' foresight in fall prevention

“Nurses should develop foresight—identifying high-risk factors or behaviors, such as patients attempting to get out of bed independently, and proactively addressing them. This ability is crucial.” (N10)

## 4 Discussion

This study revealed the core cognition and practice characteristics of rehabilitation specialist nurses in fall management through qualitative analysis. The findings showed that these nurses have a high level of alertness to fall risks and can quickly identify high-risk patients through clinical experience and professional judgment. Notably, nurses generally advocate for the establishment of a multidimensional risk assessment framework that integrates physiological functions (e.g., balance ability), psychological states (e.g., fear of falling), medication effects, and environmental factors.

### 4.1 Rehabilitation specialist nurses possess sensitivity in fall management

Our findings indicate that rehabilitation specialist nurses are sensitive to fall risks and recognize the importance of continuous learning to improve fall management. Enhancing nurse education and training is critical to improving fall management quality. Existing literature further suggests that incorporating global health (GH) concepts into rehabilitation curricula can strengthen nurses' cultural sensitivity and structural competencies (23). For example, a Canadian curriculum reform emphasized rehabilitation needs in low-income countries, fostering innovative practices in resource-limited settings (23). Additionally, simulation training and case-based discussions can improve nurses' ability to manage complex cases, such as patients with multimorbidity (24, 25). These findings underscore that recognizing fall risks and converting complex clinical information into timely preventive actions should be fundamental learning outcomes in rehabilitation nursing education. Undergraduate and postgraduate programs should explicitly integrate fall risk assessment, global health perspectives, and culturally responsive care into structured modules supported by simulation-based training and case discussions that reflect real-world multimorbidity and resource limitations. Continuing professional development for rehabilitation nurses should also provide regular updates on emerging technologies and evidence-based fall prevention strategies to maintain and strengthen these competencies.

Our participants also highlighted the importance of nurse-led rehabilitation training in preventing falls and reducing their consequences. Building on this, the broader literature points out that nurses can serve as a vital link in implementing technology-driven interventions, including virtual reality (VR)-based and gamified rehabilitation, as well as tele-rehabilitation and mobile health. For instance, VR and augmented reality (AR) technologies have been reported to provide new methods for fall prevention. Xbox Kinect–based systems can simulate real-world scenarios to help patients practice balance and gait, with immediate feedback that improves learning outcomes (6). However, prior research indicates that the widespread adoption of these technologies is limited by their cost and the need for technical support, and some older patients may resist using them because they find them difficult to operate (8).

Similarly, the expansion of nurse-led tele-rehabilitation and mobile health have advanced home-based fall management reported in the literature. The COVID-19 pandemic accelerated the adoption of tele-rehabilitation, with nurses guiding patients through home exercises via video and monitoring progress using mobile applications (e.g., fall diaries or reminder functions) (16). However, remote models cannot fully replace in-person balance assessments, and network instability may disrupt intervention continuity (16, 26).

### 4.2 Nurses should conduct comprehensive risk assessments to prevent patient falls

Our participants emphasized that fall prevention requires not only individual nursing actions but also systematic involvement and support at the organizational and interdisciplinary levels. Nurses should conduct multidimensional evaluations, as falls are influenced not only by physiological factors (e.g., age, balance, medication use) but also by psychological, environmental, and social support dimensions (27–30). Thus, assessments should extend beyond traditional physiological indicators to include cognitive status, emotional changes, family support, and ward safety (27, 31–35). Nurses frequently overlook psychological dimensions, focusing instead on visible physical impairments (35).

Advances in technology and intelligent monitoring devices have enriched biophysical parameter tracking, improving the accuracy and sensitivity of fall risk assessments. For example, photoplethysmography (PPG) sensors can continuously measure heart rate variability (HRV) via the wrist or earlobe, effectively predicting fall risks (36). AI-enhanced wearable sensors further improve predictive accuracy and promote behavioral changes through real-time biofeedback (37–39). Nursing practice should prioritize training to enhance multidimensional assessment capabilities and integrate fall risk dimensions into routine care workflows to ensure patient safety.

### 4.3 Nurses should undertake systematic involvement in fall prevention management

Our participants emphasized that fall prevention requires not only individual nursing actions but also systematic involvement and support at the organizational and interdisciplinary levels. Interdisciplinary collaboration and policy support are key success factors. For example, the iSOLVE project by Clemson et al. (40) improved fall risk assessment rates (OR=5.58) by integrating general practitioners and rehabilitation teams, though challenges remain in addressing fragmented primary care resources (41). Multidisciplinary involvement positively impacts fall management, with pharmacists reviewing medications (e.g., anticholinergic adjustments), physiotherapists conducting balance training, and clinical nurses assessing environmental risks Tiedemann et al.'s (42) fall prevention education program significantly enhanced professional knowledge and prescription behaviors, underscoring the necessity of continuous education in clinical practice.

Advanced practice nurses play a pivotal leadership role in interdisciplinary teams, with their coordination skills essential for developing personalized fall prevention plans (43). Clinical decision support systems and electronic platforms have demonstrated efficacy in fall management. For instance, real-time alerts for medication-fall risk associations (e.g., benzodiazepine dosage adjustments) have proven beneficial (44). An Australian geriatric ward case study (45) reported a 62% reduction in intervention response time after implementing an electronic fall reporting system. Thus, patient fall management should adopt a multidisciplinary, multi-system, and multidimensional approach to enhance predictive accuracy and reduce fall risks. The research findings offer valuable guidance for advancing rehabilitation nursing education. Interprofessional collaboration, systems thinking, and leadership in care coordination should be clearly integrated into the competency framework for rehabilitation nurses to strengthen their proactive and coordinating roles in multidisciplinary fall prevention programs.

### 4.4 Early assessment of fall management effectiveness is a crucial part of management

Rehabilitation specialist nurses emphasize dynamic monitoring and closed-loop feedback to optimize fall management processes, such as periodic reviews of fall event data and intervention adjustments, aligning with the “real-time response” principle in healthcare quality improvement (46). Technological advancements have introduced new assessment tools and intelligent systems, improving evaluation accuracy. Technology-driven management models, such as those using real-time monitoring of vital signs, AI-based risk predictions, and feedback-driven interventions are shifting the approach from simply reacting to falls to actively preventing them. This forward-looking approach strengthens the overall effectiveness of fall prevention efforts (36, 47, 48).

Establishing clear workflows and checklists is essential to ensure nurses' assessments are both effective and timely, especially in acute care. For instance, Johnston et al. (49) introduced a 14-item fall prevention checklist. This significantly boosted nurses' adherence to key tasks, like setting bedside alarms, and, notably, resulted in no falls during the pilot phase. Avanecean et al.'s (50) systematic review further supports that personalized care plans combined with education reduce fall rates (*p* < 0.04), though interventions should incorporate continuous monitoring and dynamic adjustment mechanisms. The use of multiple measures and tools enhances the frequency and quality of fall management effectiveness assessments, encouraging multidisciplinary engagement in fall prevention and outcome feedback.

## 5 Limitations

This qualitative study was based on a small sample size, which may limit the extent to which the findings can be applied to broader settings. Future research should include larger sample sizes and consider using mixed-method approaches to validate these findings further. Additionally, understanding the perceptions and needs of patients and their families regarding fall management would offer a richer foundation for creating effective interventions. Research is needed to assess the long-term impact of fall management strategies on patient outcomes.

## 6 Conclusions

Rehabilitation specialist nurses are crucial in fall management, and their cognition and practice directly influence patient recovery. This study highlights their professional strengths, but also points out potential challenges. Through technological innovations, educational training, and interdisciplinary cooperation, the effectiveness of fall management can be significantly improved. Future research should further explore the cost-effectiveness of personalized intervention strategies and promote policy support to optimize the accessibility and quality of rehabilitation services. Achieving the goal of “zero falls” requires the collective efforts of nurses, patients, families, and policymakers.

## Data Availability

The raw data supporting the conclusions of this article will be made available by the authors, without undue reservation.
